# Long-Term Results after Chiari Pelvic Osteotomy in the Skeletally Immature and the Role of the Anti-Chiari Effect

**DOI:** 10.3390/children10101593

**Published:** 2023-09-24

**Authors:** Eleonora Schneider, Marie-Christine Lutschounig, Klemens Vertesich, Markus Schreiner, Philipp Peloschek, Daniel Bork, Reinhard Windhager, Catharina Chiari

**Affiliations:** 1 Department of Orthopaedics, University Clinic of Orthopaedics and Trauma Surgery, Medical University of Vienna, Waehringer Guertel 18-20, 1090 Vienna, Austria; marie-christine.lutschounig@meduniwien.ac.at (M.-C.L.); klemens.vertesich@meduniwien.ac.at (K.V.); markus.schreiner@meduniwien.ac.at (M.S.); reinhard.windhager@meduniwien.ac.at (R.W.); catharina.chiari@meduniwien.ac.at (C.C.); 2 Radiology Center Vienna, Lazarettgasse 25, 1090 Vienna, Austria; ph@radiology-center.at; 3 Department of Orthopaedics and Trauma Surgery, University Clinic Knappschaftskrankenhaus Bochum, In der Schornau 23-25, 44892 Bochum, Germany; daniel.bork@kk-bochum.de

**Keywords:** Anti-Chiari effect, pelvic osteotomy, skeletally immature, long-term results, acetabular development, hip dysplasia

## Abstract

Several authors observed a loss of correction after performing Chiari pelvic osteotomy (CPO) in young patients. Hence, the aim of this study was to answer two questions: (1) Does the Chiari pelvic osteotomy affect the development of the acetabulum in skeletally immature patients in the long term? (2) Is there any evidence of the previously described “Anti-Chiari” effect after a mean follow-up of 36 years? Data from 21 patients (27 hips) undergoing CPO before the age of 16 years were clinically assessed, and the evolution of radiological parameters over time was analyzed. The mean age at CPO was 11.2 years (±3; 4.4–15.7). The 20- and 30-year survival rates of the CPO were 100% and 92.6%, respectively. Mean postoperative medialization was 54% (±18; 23–99). The average osteotomy angle was 11° (±7; 2–28). No significant changes were found for the center-edge angle (CEA) and acetabular index (AI) over time; the angle of Idelberger and Frank (ACM) almost reached normal values at follow-up (FU); for the acetabular-head index (AHI), a slight shift toward the initial situation could be detected. The morphology of the acetabulum remained unchanged over time. The “Anti-Chiari effect” seems to be primarily caused by insufficient coverage of the femoral head rather than damage to the apophysis due to surgery.

## 1. Introduction

Acetabular dysplasia is one of the major causes of early-onset osteoarthritis of the hip [1]. Depending on patient’s age and the congruency of the hip joint, different surgical techniques have been described to address the deficient femoral coverage [2,3,4,5,6,7]. Due to unsatisfying results of the formerly popular shelf operations, which were able to create coverage above the femoral head but failed to restore normal biomechanics, as its position was not addressed and, therefore, the physiologic lever arm of the pelvitrochanteric muscles was not recovered, Karl Chiari developed his pelvic osteotomy (CPO) at the Vienna University Clinic of Orthopaedics in 1952 [8]. The method’s focus lies on the medial displacement of the hip joint by performing an osteotomy right above the acetabulum. Load is then transmitted via the capsule and labrum, which are to be interposed between the femoral head and the newly created bony roof now covering the femoral head, thus improving biomechanics [8,9]. The CPO was previously applied in diverse indications, ranging from very young children suffering from congenital dislocation of the hip joint to older children affected with dysplasia of the hip with or without the presence of subluxation of the femoral head and up to adolescents and even adults afflicted with osteoarthritis secondary to hip dysplasia, but Chiari himself narrowed down these indications in the course of time. In 1969, Otte and, in 1979, Purath, however, claimed that a loss of correction could be observed when performing a CPO before growth arrest and coined the term “Anti-Chiari effect” for this observation [10,11]. Purath stated that the CPO would almost always involve damaging the important growth zones of the acetabulum, resulting in pathological growth kinetics in children, as the osteotomy has to be performed in the ultimate vicinity of the acetabulum and the craniolateral corner of the acetabulum. This issue would ultimately lead to the recurrence of dysplasia because of a disturbance of the growth zone at the apophysis of the acetabular rim, thus compromising the final result. Otte described two types of the so-called Anti-Chiari effect: the femoral head stays centered but the “newly” formed coverage drifts cranially, or the head stays in contact with the neo-coverage but leaves its postoperatively centered position.

Therefore, the aim of the present study was to (1) analyze the development of the hip after CPO in skeletally immature patients in the long term and (2) focus on any evidence of the presence of an Anti-Chiari effect in the original CPO patient cohort. This study represents a unique cohort, as all patients in this cohort were exclusively operated on by Chiari himself or surgeons personally trained by him, thus guaranteeing the use of the original technique, which was only modified in 1982, when two Kirschner wires were used to provide temporary internal transfixation of the osteotomy.

## 2. Materials and Methods

The study was approved by the institutional review board (Ethics Committee of the Medical University of Vienna; EK 431/2007). The approval date was 06 August 2007. We screened patient charts and radiographs from all patients who had undergone a CPO at our department between 1953 and 1986 prior to the age of 16 years old and invited them to attend a follow-up examination. The end of pelvic growth was established by Risser at stage IV, and patients who had reached stage IV at time of CPO were, therefore, excluded from our study. Of this consecutive series of 573 patients, 9 (2%) refused to participate in the study, 19 (3%) were dead, 21 (4%) lived abroad, 34 (6%) had to be excluded for neurological reasons and 298 (52%) were lost to follow-up. In total, 192 patients (34%) completed follow-up after a mean time of 42 years. Thereof, 108 patients (56%) were clinically and radiologically evaluated, and 84 patients (44%) were evaluated via a structured and standardized telephone interview. Extensive radiological analysis was performed in a subgroup of 21 patients with 27 native hips (25%) when radiographs at the time points pre-CPO, post-CPO and follow-up were available. Written informed consent was obtained from all patients.

### 2.1. Clinical Assessment

Clinical examination included a patient’s height and weight, range of motion, limping due to muscular insufficiency, pain or leg length difference and Trendelenburg’s and Duchenne’s tests. Trendelenburg’s test is a clinical sign of insufficiency of the Mm. glutei medius and minimus. While the patient performed a single leg stand on the affected side, the pelvis dropped to the contralateral side. In order to counteract the pelvic tilting due to muscular insufficiency, the torso leaned to the other side, which is described as Duchenne’s sign.

Pain levels were assessed using the numeric rating scale (NRS) [12], where patients were asked to mark one number between zero and ten best representing their intensity of pain at the time of follow-up examination. The Harris Hip Score (HHS) [13] was used to measure the outcome with regard to hip function and symptoms. A maximum score of 100 points could be achieved in healthy individuals. The interpretation of the results could be executed as follows: 90–100 points stands for excellent, 80–90 for good, 70–80 for fair and <70 for poor outcomes.

### 2.2. Radiological Evaluation

Standardized plain radiographs, including an anteroposterior (AP) pelvis, AP and lateral hip and false-profile view, were obtained from all patients at follow-up. For the AP pelvis, patients were positioned in supine position, and lower limbs were 15° internally rotated from the hip to gain an AP view of the proximal femur. The false-profile view served as an oblique view of the acetabular edge in order to quantify the anterior coverage of the femoral head, as well as arthritic affection of the anterior part of the hip joint. Preoperative and postoperative radiographs were collected from our archives, scanned and imported into our PACS system (Agfa^TM^). Radiographs were analyzed using the TraumaCad^®^ software (Brainlab^TM^, Version 2.5).

The center-edge angle (CEA), as published by Ogata [14,15]; the acetabular head index (AHI); the acetabular index depth to width (AI) of Heyman and Herndorn [16,17]; and the acetabulum (ACM) angle of Idelberger and Frank [18,19] were measured. The CEA describes the position of the femoral head in relation to the acetabulum and can be measured on an AP pelvis. The deeper the acetabulum, the higher the value of the angle. In dysplastic hips (shallow acetabula with a short roof), the values decreased. The CEA is one of the most important measures used in order to quantify the extent of dysplasia in hips joints. The AHI is used to assess femoral head coverage on plain radiographs and tends to decrease with increasing age. It is used to reveal the abnormal lateral displacement (subluxation) of the femoral head in children [17]. The AI depicts the ratio of the acetabular width, defined as the distance from the most inferior point of the acetabulum to the lateral edge of the acetabular roof, and the depth of the acetabulum on an AP pelvic view. In dysplastic hips with flat acetabula, values tend to be low. The ACM also refers to the depth of the acetabulum. The higher the values of the ACM, the more dysplastic the acetabula. Normal values are summarized in Table 1. A potential Anti-Chiari effect would be characterized by the following changes over time: a reduction in the CEA, reduced AHI and AI caused by loss of lateral coverage and decentralization of the head and an increase in the ACM due to the flattening of the acetabulum.

Medialization, as the percentage of medial displacement of the inferior pelvic fragment at the level of the osteotomy, and the quality of the osteotomy were assessed on postoperative AP pelvic radiographs. The angle of the osteotomy was assessed, and the osteotomy was then classified as ideal (10°–15°), flat (<10°), steep (>15°) or intra-articular. Osteoarthritis was categorized according to Toennis grades 0 to 3 [20,21] at the three time points (pre- and post-CPO and FU). Grade 0 stands for the absence of osteoarthritis signs in the hip joint. Grade 1 would show a slight narrowing of the joint space, slight lipping at the joint margin and slight sclerosis of the femoral head or acetabulum. The presence of small cysts and moderate loss of sphericity of the femoral head would be classified as grade 2. The highest score of three would be marked by the radiographic appearance of large cysts, avascular necrosis or severe deformity of the femoral head and/or obliteration of the joint space. The joint congruency (centered, subluxed and dislocated) and femoral head shape (round, elliptic, aspheric and necrotic) was evaluated via the final follow-up X-rays (AP). Patients with total hip arthroplasty (THA) were excluded from radiological FU analysis.

### 2.3. Descriptive Statistics

The absolute and relative frequencies of the mentioned parameters are reported. Distribution of continuous variables was analyzed, and mean and standard deviation for normally distributed variables and the median and interquartile range for non-normally distributed variables were reported.

To compare continuous, non-normally distributed variables in two independent groups of patients the Mann–Whitney U Test were applied. Fisher’s Exact Test for dichotomous variables was chosen due to the small number of patients in one of the two subgroups. To compare continuous, non-normally distributed variables in three independent groups of patients, the Kruskal–Wallis Test was used.

Kaplan–Meier analyses [22] with conversion THA as an endpoint were performed stratified for certain characteristics. Differences between strata were tested for statistical significance via the Log-Rank test, which is a non-parametric test used to compare the survival distributions of subgroups or samples in right-censored data.

Excel^®^ (Microsoft Corporation, Redmond, Seattle, USA; version 14.3.2) was used to collect the data, and calculations were computed using SPSS^®^ (IBM, Armonk, NY, USA; version 27).

## 3. Results

### 3.1. Patient Demographics

The cohort comprised 13 female (62%) and 8 male (38%) patients with 27 CPOs. The mean age at CPO was 11.2 years (±3; 4.4–15.7). FU was performed after an average time of 36 years (±6; 25–46) after CPO. The preoperative diagnoses were developmental dysplasia of the hip (DDH) in 21 hips (78%), DDH with subluxation in 4 hips (15%) and luxation as an infant in two hips (7%). Moreover, 21 hips (78%) had received closed reduction and cast treatment prior to CPO. With respect to previous or concomitant hip surgeries, ten hips had no prior surgery (37%); in eight hips, a varus or valgus osteotomy was performed (30%); in seven (26%); open reduction of the hip was necessary; and two hips (7%) underwent a de-rotation osteotomy of the proximal femur.

**Figure 1:**
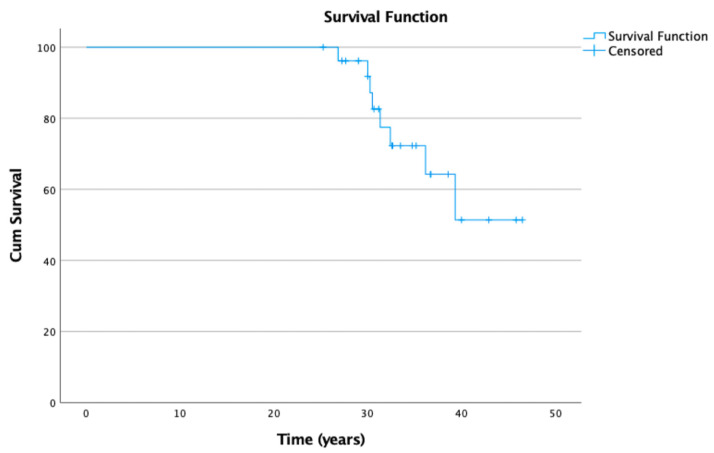
Kaplan–Meier curve for overall survival with conversion to total hip arthroplasty as an endpoint.

The overall survival of the CPO was 34 years (±6; 25–47). The 10-, 20- and 30-year survival rates were 100%, 100% and 92.6% respectively (Figure 1). At FU, six patients (8 hips; 30%) had undergone conversion surgery via THA after an average time of 32 years (±3.9; 27–39) after CPO. The average age at THA was 43 years (±3.9; 38–48). The younger the patients were at time of CPO (<11 years), the better the survival (*p* < 0.0001).

### 3.2. Clinical Results

Clinical results are summarized in Table 2. All patients presented with acceptable ROM and pain levels.

Fourteen patients (52%) presented with a clinically relevant leg length difference of more than one centimeter (cm). Eight patients (30%) were limping due to muscular insufficiency, and five (19%) patients limped due to leg length discrepancy. The Trendelenburg test was positive in eleven hips (41%), and the Duchenne sign was positive in six hips (22%). The HHS results are summarized in Table 3.

### 3.3. Radiological Results

Pre- and post-CPO radiographs from all 27 hips were assessed, whereas 19 radiographs were evaluated at FU, as all hips with conversion THA were excluded.

The mean postoperative medialization was 54% (±18; 23–99). The average osteotomy angle was 11° (±7; 2–28), and, therefore, the osteotomy was categorized as ideal in 21 hips (78%), flat in three hips (11%), intra-articular in two hips (7%) and steep in one hip (4%). None of the patients showed a descending (negative angle) or high osteotomy. There was no significant difference in patients with preserved CPO at FU compared to conversion THA with regard to medialization (*p* = 0.852).

A summary of radiological results and changes over time is depicted in Table 4.

**Figure 2:**
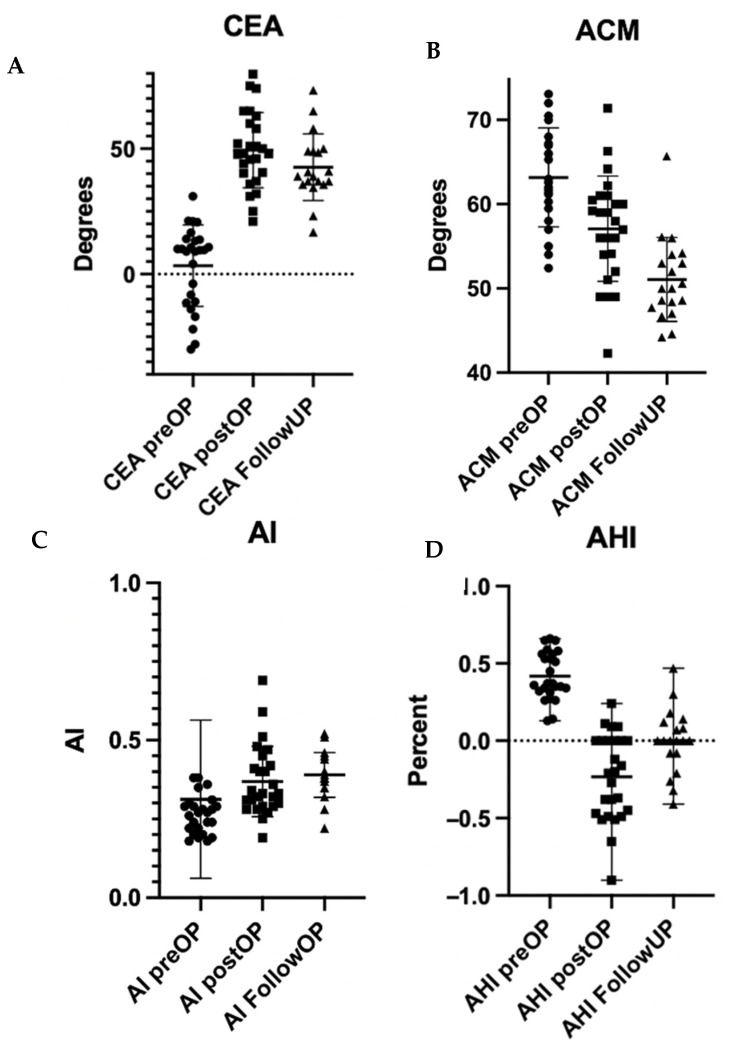
(**A**–**D**): The Boxplot depicts the comparison of the development of the angles ((**A**): CEA; (**B**): ACM, (**C**): AI; (**D**): AHI) over time.

When comparing the imaging post-CPO and at follow-up, AI remained relatively constant over time. CEA demonstrated a slight decrease, which, however, did not reach statistical significance. The acetabular head index decreased over time, demonstrating a slight shift toward the initial situation (*p* = 0.008). The ACM significantly decreased over time, reaching almost normal values at FU. The changes over time are depicted in Figure 2.

Concerning osteoarthritis, according to Toennis, 25 hips presented with grade 0 (93%) and two hips presented with grade 1 (7%) at pre- and post-CPO evaluation. At FU, two hips were grade 1 (11%), eight hips were grade 2 (42%) and nine hips were grade 3 (47%).

All patients had a congruent hip joint at FU evaluation.

The shape of the femoral head was classified as round in one (AP; 5%) and seven hips (false-profile; 37%), elliptic in eleven (AP; 58%) and nine hips (false-profile; 47%), aspheric in six (AP; 32%) and two hips (false-profile; 11%) and necrotic in one hip (AP and false-profile; 5%).

**Figure 3:**
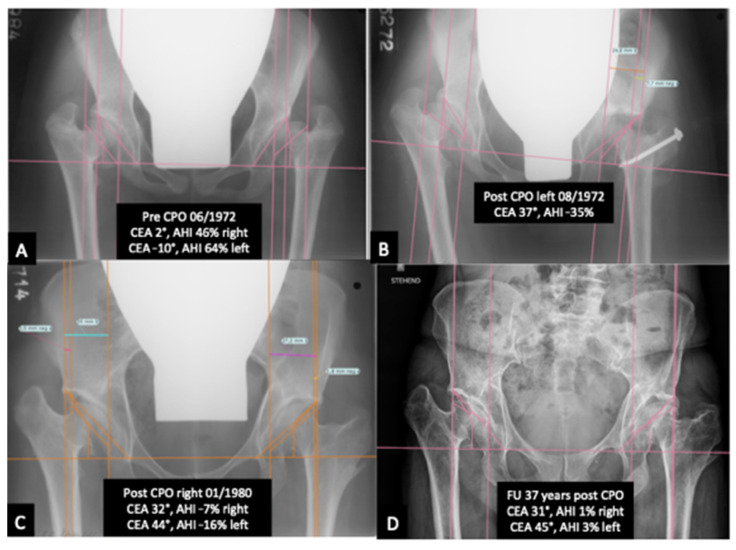
(**A**–**D**): An exemplary case is shown in Figure 3. (**A**) depicts an ap pelvis radiograph prior to CPO. (**B**) shows the postoperative result of the left hip. (**C**) displays the postoperative result of the right hip. (**D**) illustrates the long-term results 37 years after CPO.

Regarding the influence of pre-CPO CEA on THA, no significance could be reached, but a trend toward THA for lower CEA levels preoperatively could be noticed (*p* = 0.09). This trend was not the case for CEA levels post-CPO (*p*= 0.129). An exemplary case is shown in Figure 3.

## 4. Discussion

Chiari’s pelvic osteotomy was the first surgical procedure to address the problem of hip dysplasia by completely cutting the iliac bone and changing the position of the acetabulum via the medialization of the distal fragment. When the surgery is performed prior to growth arrest, Otte and Purath have described the so-called “Anti-Chiari effect”. They stated that a loss of correction could be observed when performing a CPO before reaching skeletal maturity. Otte hypothesized that the primary pathological development of the acetabulum due to DDH combined with the osteotomy would lead to recurring dysplasia of the hip joint. Purath assumed that a direct lesion of the acetabular growth zone was causal.

Due to controversy about how performing a CPO in children affects the further development of the hip, we evaluated the results at our institution regarding overall survival of CPO in children, radiographic results and changes over time, as well as pain levels after 36 years.

In this series, the majority of CPOs were performed by Chiari himself (N = 17; 63%). Even though we could observe excellent long-term results, half of the patients had a clinically relevant leg length difference, and 30% presented an impaired gait at FU examination, but intensive physiotherapy and strengthening of the abductor muscles can diminish limping. In general, ROM was acceptable, but an average flexion of 89° ± 19 (40–125) in the concerned hip joint supports the clinical rule that patients with a preoperative flexion of less than 90° should not be operated on, as ROM tends to deteriorate after CPO. Pain levels remained stable over the observation period, and NRS 2 was the average pain level at follow-up examination.

The average preoperative CEA was 3°, while the postoperative CEA was 50° and that at FU 36 years later was 43°. Only one hip had a CEA lower than 25° (namely 21°) after the CPO. Postoperative medialization was 54% on average. The radiological measurements showed no evidence of the Anti-Chiari effect as loss of coverage and decentralization of the femoral head could be ruled out.

In the literature, controversial results can be found. Rejholec et al. [23] analyzed the long-term effectiveness of the CPO and varus femoral osteotomy in the treatment of DDH in 1991. He concluded that CPO, even when combined with femoral varus osteotomy, is followed by recurrent valgus deformity and a positive Anti-Chiari effect, especially when performed before the age of eight years old.

In 2002, Osebold et al. [24] observed in one out of ten hips (10%; average age at surgery eleven years; 5–19) in patients with neuromuscular disease an Anti-Chiari effect after a mean FU of ten years (6–11).

More recently, Karami et al. referred to the CPO in 2008 as the most controversial osteotomy of the hip joint [25]. He observed 20 hips after CPO (mean age at surgery 12.6 years; 9–18) for 54 months. The average osteotomy angle was 12°, and the mean displacement was 42%. He concluded that CPO could produce good-to-excellent results in adolescent patients with specific indications, including painful dysplasia or uncoverage of the femoral head, in which incongruity or poor acetabular development render other reconstructive procedures inappropriate.

In 2011 Vukasinovic et al. [26] assessed 99 hips after CPO (mean age at surgery 15.6. years; 10–19) and found an average osteotomy angle of 13°, mean medialization of 43% and an improvement in the CEA from 10° preoperatively to 39° at FU. He postulated that the CPO has retained its leading position in the treatment of adolescent hip disorders, despite the availability of biologically superior procedures nowadays.

However, direct comparisons of data are difficult as most studies included patients up to 19 years of age at time of surgery and, therefore, partly skeletally mature patients.

Our study has limitations, as it is a retrospective, single-center study without a control group. Additionally, retrospectively analyzed data have several potential limitations, such as incompleteness and issues with the integrity of the collected data, selection bias and loss to follow-up. Furthermore, the patient cohort at FU is small and statistical evaluation is, therefore, difficult and possibly insufficient to identify a small Anti-Chiari effect.

In this cohort, the CPO was performed in children as young as four years of age and performed best in children below the age of eleven years old. From today’s perspective, however, the reorientation of the acetabulum via the Dega [4] or Pemberton [3] type of osteotomies is to be preferred in toddlers, and the Salter osteotomy [2] or triple osteotomy [6] are typically used in school-age children. Those reorientation osteotomies, which were developed after the CPO and serve as today’s gold standard, are deemed to have more potential for correction, and the weight bearing area remains covered by articular cartilage. Nowadays, the CPO is reserved for salvage cases of severe dysplasia with the lateralization of the femoral head and incongruent joints, as seen after Perthes disease or avascular necrosis of the femoral head, and is rarely used in children below the age of eight years old. Nevertheless, osteoarthritis was expected to occur rather early after performing CPO, as the interposed capsule and labrum transform into fibrocartilage over time, having inferior properties compared to the hyaline cartilage. But the sufficient medialization and, thus, stabilization of the femoral head by creating a stable bony roof seem to outweigh the possible disadvantages of fibrocartilage in the load transmission area. Windhager et al. [27] and Lack et al. [28] have already emphasized earlier that sufficient medialization is the most important criterion for successful correction and good long-term results. Our data support this statement, as we did not observe an Anti-Chiari effect, which would have led to a loss of femoral head coverage as skeletal growth continued after CPO. Actually, it seems as if long-term results of the reorientation osteotomies have not yet surpassed the survival rates of the CPO, despite their advantages. In 2017, Lerch et al. matched the long-term results with THA as an endpoint of the periacetabular osteotomy (PAO), the triple osteotomy, the rotational osteotomy and the CPO with overall survival of THA (with revision surgery as an endpoint) in female patients below the age of 50 years old [29]. A continuous decrease in survival could be observed for all types of osteotomies, as well as the THA, over 30 years. It revealed similar or even better survival of the CPO compared to the other procedures. We recently published a report of up to 40-year overall survival of 504 hips after CPO [30]. After an average follow-up of 37 years, osteoarthritis has significantly increased with 53% Toennis grade 3 but the cumulative survival was 80% at twenty years, 57% at thirty years and 35% at forty years. Furthermore, we showed that precedent CPO in dysplastic hips does not compromise the requisite for successful conversion THA at a later stage [31].

## 5. Conclusions

The results support the overall effectiveness and durability of the Chiari pelvic osteotomy in skeletally immature patients. The “Anti-Chiari effect” seems to be primarily caused by insufficient coverage of the femoral head at the index surgery rather than damage to the apophysis during surgery and subsequent loss of correction. Hence, the CPO is still of justified importance to certain indications. Accurate patient selection and precise adherence to the technical details of the surgery will lead to a successful outcome, even when performed prior to skeletal maturity.

## Figures and Tables

**Table 1:** Summary of normal values.

Parameter	Normal Value
CEA	>25° ^a^
ACM	>2 years: <50° ^b^
AHI	2–14 years 94% ^c^
AI	>0.34

CEA: center-edge angle, ACM: angle for acetabular depth, AHI: acetabular head index, AI: acetabular index depth to width. ^a^ [15], ^b^ [19] and ^c^ [17].

**Table 2:** Summary of Clinical Results (N = 27).

Clinical Evaluation	Flexion ^a^	Abduction ^a^	Adduction ^a^	Outward rotation ^a^	Inward rotation ^a^	NRS	BMI
Results	89 ± 19 (40–125)	37 ± 14 (10–70)	32 ± 11 (10–50)	25 ± 16 (0–50)	18 ± 14 (0–50)	2 ± 2.4 (0–6.9)	25 ± 4 (19–32)

CPO—Chiari pelvic osteotomy, NRS—numeric rating scale, BMI—body mass index as in kg/m^2^; ^a^ values given in degrees, and results given as mean ± standard deviation (range).

**Table 3:** Results of the Harris Hip Score (N = 21).

HHS Domain	Total	Pain	Function
Results	86 ± 15 (49–100)	37 ± 10 (10–44)	42 ± 6 (24–47)

Values given as mean ± standard deviation (range).

**Table 4:** Summary of radiological results.

	Pre-CPO (N = 27)	Post-CPO (N = 27)	FU (N = 19)	*p* Value
CEA	3° (±16; −30–31)	50° (±15; 21–80)	43° (±13; 17–73)	0.110
ACM	63° (±6; 52–73)	57° (±6; 42–71)	51° (±5; 44–66)	<0.001
AI	0.3 (±0.3; 0.2–1.5)	0.4 (±0.1; 0.2–0.7)	0.4 (±0.07; 0.2–0.5)	0.206
AHI	42% (±16; 13–66)	123% (±28; 0–189)	110% (±20; 0–140)	0.008

*p*-value was calculated for comparison between values post-CPO and at FU. CPO Chiari pelvic osteotomy; FU, follow-up; CEA, center-edge angle; ACM, angle of Idelberger and Frank; AI, acetabular index of depth to width of Heyman and Herndorn; AHI, acetabular head index.

## Data Availability

The data presented in this study are available on request from the corresponding author. The data are not publicly available due to privacy concerns.
